# Recent advances in the development of histone deacylase SIRT2 inhibitors

**DOI:** 10.1039/d0ra06316a

**Published:** 2020-10-09

**Authors:** Wenyu Yang, Wei Chen, Huilin Su, Rong Li, Chen Song, Zhouyu Wang, Lingling Yang

**Affiliations:** College of Food and Bioengineering, Xihua University Chengdu 610039 China; College of Science, Xihua University Sichuan 610039 China yangll0808@sina.com +86-28-87720552

## Abstract

Sirtuin 2 (SIRT2) is an important and special member of the atypical histone deacetylase Sirtuin (SIRT) family. Due to its extensive catalytic effects, SIRT2 can regulate autophagy, myelination, immunity, inflammation and other physiological processes. Recent evidence revealed that dysregulation of human SIRT2 activity is associated with the pathogenesis and prognosis of cancers, Parkinson's disease and other disorders; thus SIRT2 is a promising target for potential therapeutic intervention. This review presents a systematic summary of nine chemotypes of small-molecule SIRT2 inhibitors, particularly including the discovery and structural optimization strategies, which will be useful for future efforts to develop new inhibitors targeting SIRT2 and associated target proteins.

## Abstract 2

This review presents a systematic summarization of nine chemotypes of small-molecule SIRT2 inhibitors, which will be useful for future efforts to develop new inhibitors targeting SIRT2 and associated target proteins.

## Graphical abstract

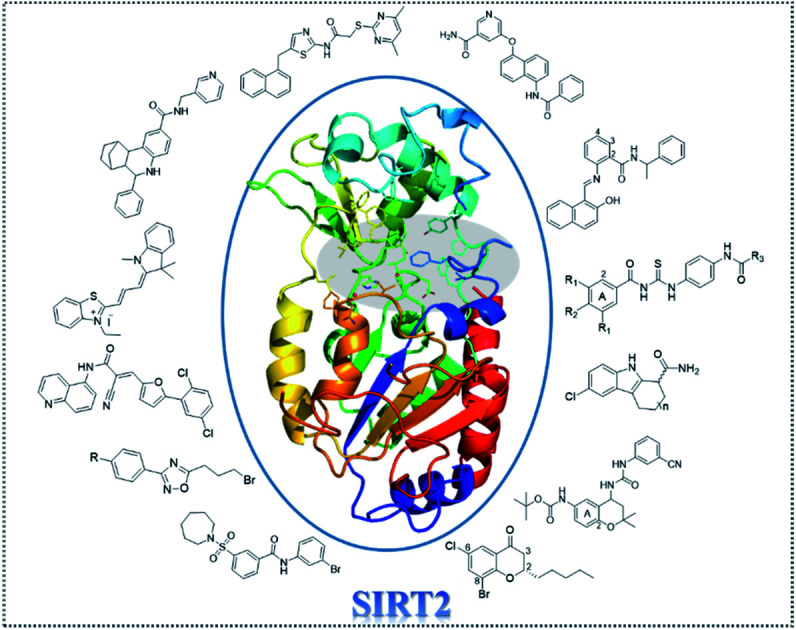

## 1. Introduction

The human genome encodes seven different sirtuin isotypes, namely SIRT1-7, which belong to the atypical histone deacetylase family.^1^ The major function of SIRTs is to cleave off acetyl groups and other acyl groups from the ε-amino group of lysines in histones or other substrate proteins in the presence of the cofactor NAD^+^.^2–5^ SIRT1–7 have different subcellular localizations and functions.^3^ SIRT2 is the only SIRT member that is mainly distributed in the cytoplasm but shuttled to the nucleus during mitosis.^6^ Several studies revealed that SIRT2 catalyzes the deacetylation modification of histones H3K18, H3K56, H4K16, *etc.*, so that histones tightly bind to negatively charged DNA, resulting in dense curling of chromatin and ultimately suppression of gene transcription and expression.^7,8^ In addition, a multitude of non-histone substrates of SIRT2, such as α-tubulin, P300, FOXO1/3a, HIF-1α, eIF5A, CDH1, NF-κB, and PKM2, have been identified in recent years.^3,8–11^ Recent crystallographic analyses revealed that the active site of SIRT2 has a large hydrophobic pocket that can accommodate long-chain fatty acyl groups, thereby catalyzing the removal of long-chain fatty acyl groups.^12^

By interacting with various substrates, SIRT2 has been implicated in a wide range of cellular processes such as cell cycle regulation, autophagy, myelination, neurodegeneration, glucose metabolism, and inflammatory response.^8,10,13,14^ Recent literatures revealed the important roles of SIRT2 in the pathogenesis, development and prognosis of various diseases.^3,15^ For example, Chen *et al.* found that SIRT2 is highly expressed in hepatocellular carcinoma (HCC) cell lines and human HCC tissues, which contributes to cell motility and invasiveness of HCC cells.^16^ The upregulation of SIRT2 in primary HCC tumors is significantly associated with more advanced tumor stages, and shorter overall survival time. Jing *et al.* reported that SIRT2 can block the degradation of the oncoprotein *c*-Myc, and the potent and specific SIRT2 inhibitor TM can effectively promote the ubiquitination and degradation of *c*-Myc, showing broad inhibitory effects against a variety of human cancers *in vitro* and *in vivo*.^17^ Xu *et al.* found for the first time that SIRT2 expression in relapsed acute myeloid leukemia (AML) patients is higher than that of newly diagnosed patients, and SIRT2 is involved in multidrug resistant AML mainly *via* ERK1/2 signaling pathway.^18^ Hoffmann *et al.* found that selective SIRT2 inhibitors AEM1 and AEM2 can increase the expression of cell cycle-related gene CDKN1A and pro-apoptotic genes PUMA and NOXA by up-regulating the acetylation level of p53, thereby resulting in increased sensitivity of non-small cell lung cancer cells A549 and H1299 to the chemotherapy agent etoposide.^19^ In addition, Outeiro *et al.* found that inhibition of SIRT2 in the Parkinson's cell model not only reversed the α-synuclein-mediated toxicity, but also protected dopaminergic nerves from necrosis *in vitro* and in the Drosophila Parkinson's model, thereby relieving Parkinson's symptoms.^15,20^ These studies clearly show that SIRT2 is a potential drug target for associated diseases.

**Fig. 1:**
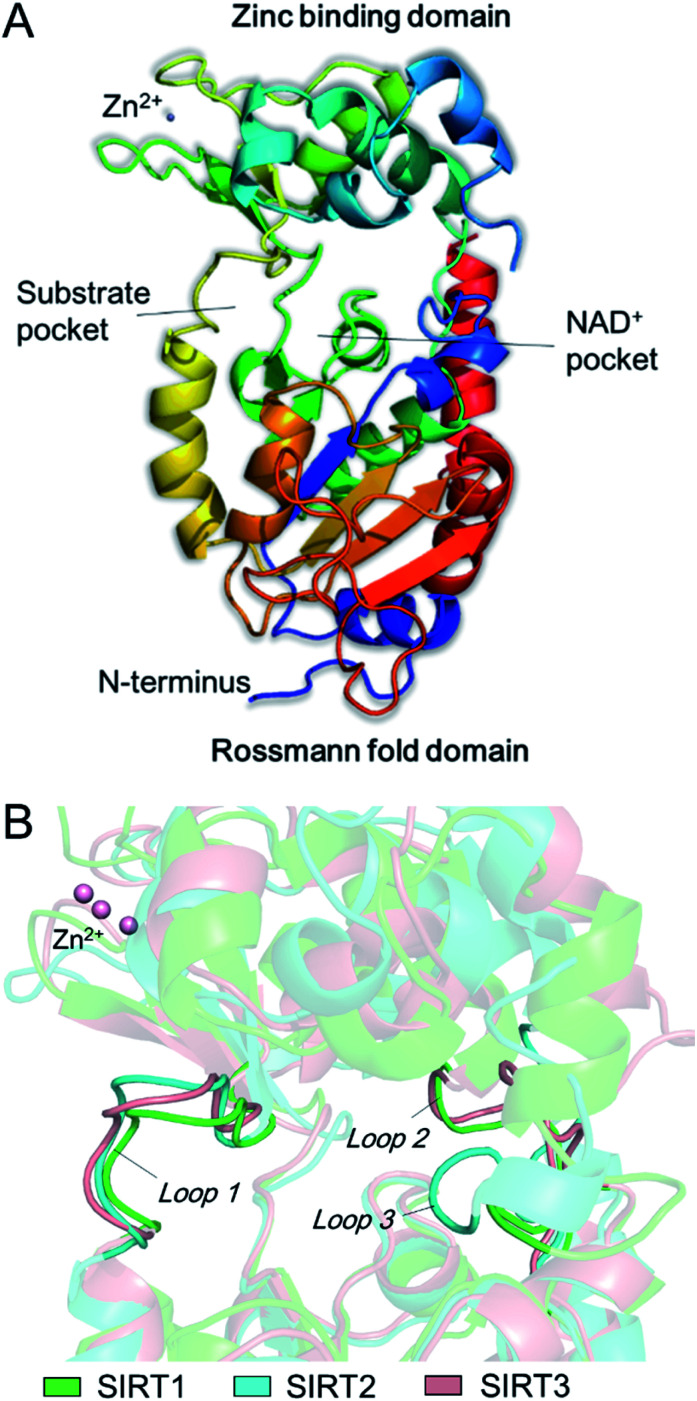
(A) The structure of SIRT2 protein. (B) Observed differences between the loops around the acyl-lysine substrate.

The catalytic core of SIRT2 contains two main domains: a larger domain that is a Rossmann fold NAD^+^ binding pocket, consisting of 6 β-strands (β1-3 and β7-9) and 6 α-helixes (α1, α7, α8, and α10-α12), and a small domain that contains a structural zinc ion (Fig. 1A). These two domains are connected by four crossovers that form a large groove. There is a specific pocket in the active site, lined with a number of hydrophobic residues, intersects the groove, indicating it may be a class-specific binding site to recognize its specific substrates. The large groove contains the NAD^+^ binding site and the residues that are conserved among sirtuins (Fig. 1A). Evidences showed that mutation of this site will result in the loss of deacetylation of catalytic activity, indicating this groove is the catalytic core. In addition, there are 19 residues in the N-terminal extension of SIRT2 from an amphipathic α-helix that has no contacts with the protein, suggesting the N-terminal extension is not essential for SIRT2 catalytic activity, while this amphipathic helixes may have an important contribution to protein–protein interactions in transcriptional regulation.^21^ In addition, by comparison of SIRT1, SIRT2 and SIRT3, we observed substantial differences between the loops that can form different hydrophobic binding sites. Uniquely, SIRT2 has a long hydrophobic pocket which is able to accommodate long-chain fatty acyl groups as revealed by crystallographic analyses,^22^ and also represent specific structural characteristics for inhibitor development.

## 2. Inhibitor development

As the roles of SIRT2 in different diseases have been gradually revealed, SIRT2 inhibitors are considered as potential effective molecules for the treatment of related diseases.^23^ Currently reported SIRT2 inhibitors can be divided into two categories: peptide substrate-modified^24–26^ and small-molecule inhibitors. Considering the possible poor drug-like properties of peptide inhibitors, such as undesirable physicochemical properties and metabolic instability, this review mainly focused on structurally different small-molecule SIRT2 inhibitors, with the aim to provide substantial information for the development of specific small molecules targeting SIRT2.

### 2.1 SirReal2 and its analogues

**Fig. 2:**
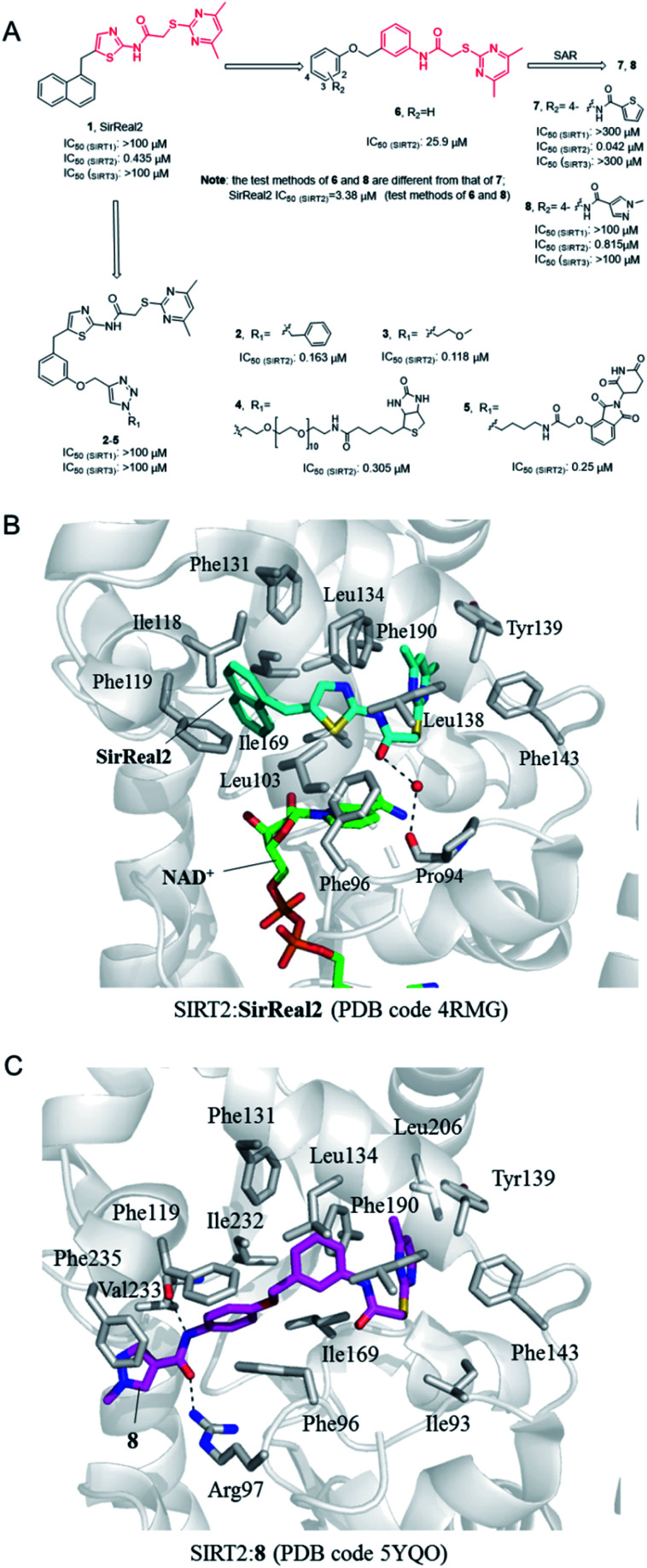
(A) Chemical structures of SirReals-based SIRT2 inhibitors and their inhibitory activity data on SIRTs obtained by fluorescence-based methods.^29–31^ (B and C) Crystallographic analyses revealed the binding features of SirReal2 and 8 with SIRT2.

In 2015, Rumpf *et al.* discovered a class of SIRT2 allosteric inhibitors, represented by SirReal2 (1, Fig. 2A).^27^ These inhibitors show high potency and selectivity to SIRT2 due to their ability of inducing active-site rearrangement of SIRT2. The SIRT2:SirReal2 structure (4RMG) reported by this group is the first complex structure of SIRT2 protein with a selective small-molecule inhibitor, revealing that SirReal2 binds to the induced hydrophobic pocket and mainly make hydrophobic interactions with a set of hydrophobic residues (Fig. 2B). This crystal structure provided important basis and insights into the high selectivity of SirReal2, and suggested that the deep hydrophobic pocket is unique for the design of selective SIRT2 inhibitors. Subsequently, guided by the interaction features obtained from SIRT2:SirReal complex, the same group designed and synthesized compounds 2 and 3, which showed better potency, water solubility, and cellular efficacy; they also developed a SIRT2-selective affinity probe (4, Fig. 2A), providing an important chemical tool for further exploration of SIRT2 biology.^28^ Further, they developed an effective proteolytic targeting chimera (PROTAC) inhibitor 5 by rationally linking the thalidomide ligand with SirReal inhibitors, providing another tool for expanded pharmacological researches by chemically induced SIRT2-knockout.^29^

Recently we used SirReal2 as a lead compound to develop new SIRT2 inhibitors with the aid of lead optimization program, LEADOPT.^30,31^ By replacing the thiazole ring in SirReal2 with benzene ring, a set of *N*-(3-(phenoxymethyl)phenyl)acetamide derivatives were developed as selective SIRT2 inhibitors, as, for example compound 6. Systematic structure–activity relationship (SAR) studies of SIRT2 inhibitors with 2-((4,6-dimethylpyrimidin-2-yl)thio)-*N*-phenylacetamide core scaffold led to the discovery of more potent inhibitors such as 7 and 8.^30,31^ Crystal structure revealed the specific hydrophobic binding features of 8 to SIRT2 (Fig. 2C), which likely mimics the binding of long-chain acyl-lysine substrates.^27^ Biological experiments have shown that compound 7 has obvious inhibitory effects on breast cancer cell MCF-7 and can increase the acetylation level of α-tubulin in a dose-dependent manner. Moreover, compound 8 is a potent SIRT2 inhibitor and can effectively inhibit the growth, invasion and migration of non-small cell lung cancer (NSCLC) cells, providing more references and options for the research of SIRT2 targeted cancer treatment.^30^

### 2.2 Nicotinamide and its analogs

**Fig. 3:**
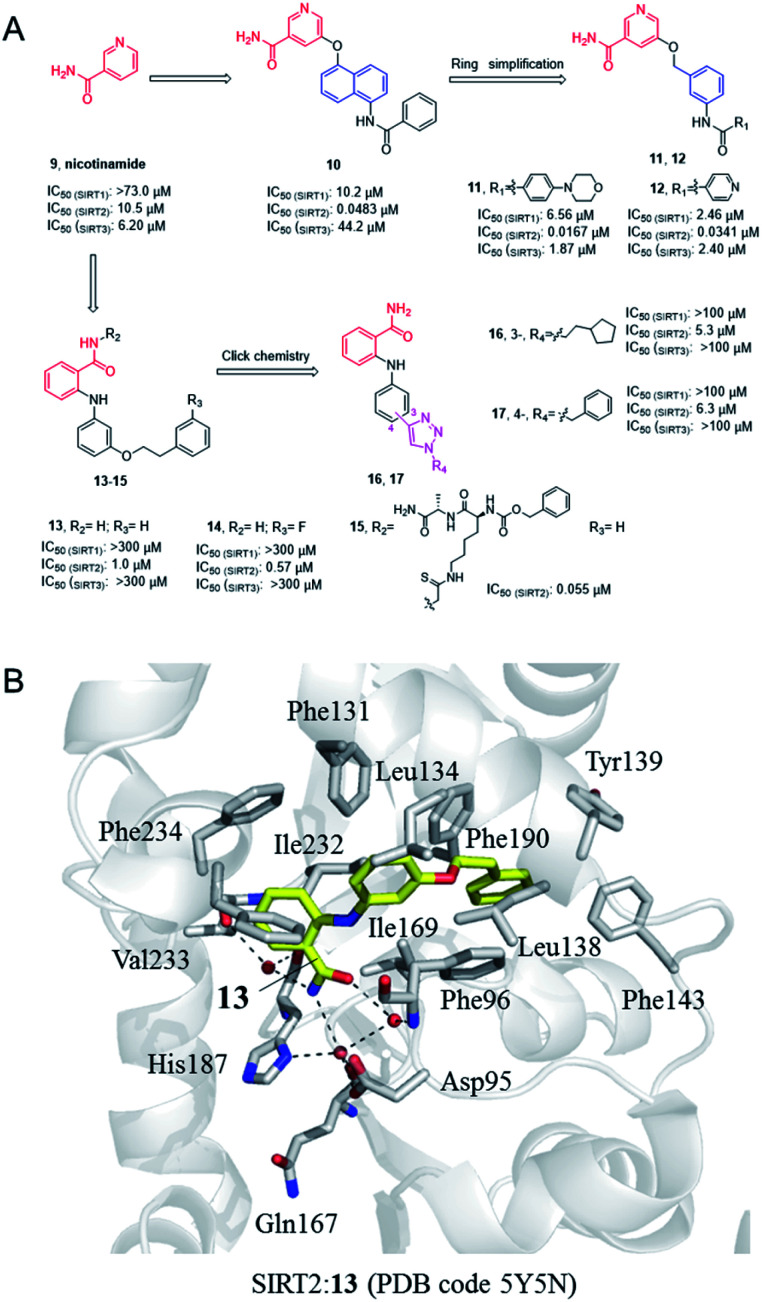
(A) Chemical structures of nicotinamide-based SIRT2 inhibitors and their inhibitory activity data on SIRTs determined by Fluor de Lys assays.^34^ (B) Crystallographic analyses revealed the binding features of 13 with SIRT2.

Nicotinamide (9, Fig. 3), as a product of sirtuins-catalyzed deacetylation reaction, is a weak and non-selective natural inhibitor of sirtuins, mainly inhibiting the deacetylation course.^32^ Due to its weak SIRT2 inhibitory activity, Cui *et al.* conducted a systematic SAR study on nicotinamides through a fragment-based approach, *i.e.* the naphthylamide sulfonic acid scaffold of SIRTs inhibitor suramin and nicotinamide scaffold, leading to the discovery of aromatic formamide naphthalene–nicotinamide derivatives. Among them, compound 10 (Fig. 3) exhibits potent SIRT2 inhibition with IC_50_ values of 48.3 nM, and remarkable selectivity for SIRT2 over highly homologous SIRT1 and SIRT3.^33^ The *in vitro* cell-based assays showed that compound 10 can increase the acetylation level of α-tubulin in both concentration- and time-dependent manners. Kinetic studies have shown that compound 10 acts as an acetyl-lysine substrate competitive inhibitor, rather than an NAD^+^ competitive inhibitor. Derived from the 5-aminonaphthalen-1-yloxy nicotinamide core scaffold, a series of new 5-((3-amidobenzyl)oxy)-nicotinamide SIRT2 inhibitors were reported by the same group, *e.g.*11 and 12 (Fig. 3). Compound 11 may be competitive against both substrate and NAD^+^; 11 and 12 can significantly reverse the cytotoxicity induced by α-synuclein aggregation in SH-SY5Y cells, which laid the foundation for the research of SIRT2 inhibitors for potential therapy against Parkinson's disease.^35^

In 2012, Suzuki *et al.* characterized a series of 2-anilinobenzamide analogues containing nicotinamide-like benzamide as SIRT2 inhibitors and conducted relatively SAR studies which led to compounds 13 and 14 as potent SIRT2 inhibitors (Fig. 3A). Compared with the previously reported selective SIRT2 inhibitor AGK2, 13 and 14 showed better inhibitory activity on SIRT2, and had higher selectivity for SIRT2 over SIRT1 and SIRT3. The crystal structure of SIRT2:13 (PDB code 5Y5N)^34^ revealed that 13 binds to form hydrophobic contacts with the induced hydrophobic binding site, and the nicotinamide moiety is positioned to make water-bridging hydrogen-bonds with Phe96, Gln167, Asp95, and His187 (Fig. 3B), which further highlighted the importance of hydrophobic binding site to SIRT2 selective inhibition. Based on the previous SAR studies of anilinobenzamide derivatives, the same group designed and synthesized a compound library of 114 *meta*- and *para*-substituted 2-anilinobenzamides as potential SIRT inhibitors *via* click chemistry, and subsequent screening identified two SIRT2-selective inhibitors 16 and 17 (Fig. 3A), which show similar inhibition but better selectivity than AGK2.^36^ In 2017, Based on the SIRT2:13 complex structure, Suzuki’ group carried out structural modifications to the amide moiety of 2-anilinobenzamide scaffold, leading to the identification of a novel SIRT2 inhibitor 15 which occupies both the substrate-binding site (*i.e.*, the “selectivity pocket”) and the NAD^+^-binding site (Fig. 3B).^34^ Moreover, 15 exhibited apparent anti-proliferative activity in MDA-MB-231 and MCF-7 breast cancer cell lines.^34^

### 2.3 Hydroxynaphthaldehyde derivatives

**Fig. 4:**
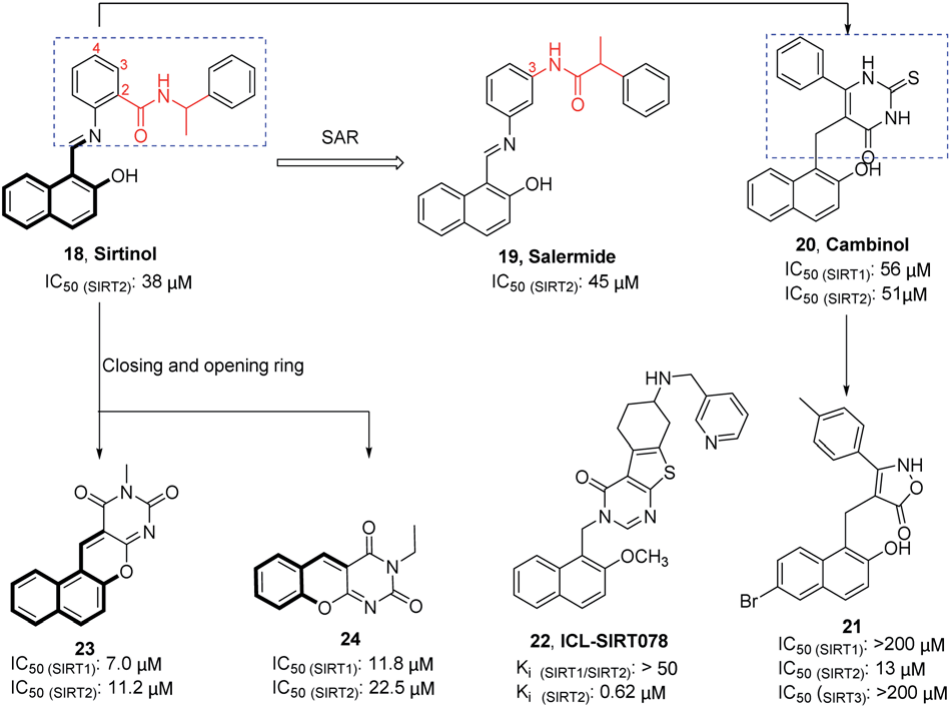
Chemical structures of hydroxynaphthaldehyde-based SIRT2 inhibitors and their inhibitory activity data on SIRTs by fluorescence-based or SIRT-Glo assays.^38–40^

Sirtinol (18, Fig. 4) is one of the earliest reported SIRT2 inhibitors. Although its weak inhibitory activity on SIRT2 and considerable inhibitory activity on SIRT1, studies have shown that Sirtinol is effective against tumors *in vitro*. As exemplified in the study by Ota *et al.*, Sirtinol induces senescence-like growth arrest in MCF-7 and H1299 cell lines.^37^ The structural optimization of the linker (red mark in Fig. 4) in the Sirtinol structure led to the discovery of Salermide (19, Fig. 4).^38^ Compared to Sirtinol, Salermide has similar inhibitory activity against SIRT2, and higher anti-proliferative activity and apoptosis induction properties in a panel of cancer cell lines. Direct replacement of substituted aniline in Sirtinol with 6-phenyl-2-thiouracil resulted in another SIRT2 inhibitor Cambinol (20, Fig. 4), which is the first reported SIRT2 inhibitor with antitumor activity *in vivo*.^41^ Cambinol induces apoptosis of BCL-6-expressing Burkitt lymphocytes and exerts antitumor activity in Burkitt lymphoma xenografts. Ongoing efforts of optimizing Cambinol by substituting the pyrimidinedione ring with other heterocycles to improve potency and selectivity resulted in 21 and 22 (ICL-SIRT078, Fig. 4); both compounds showed high selectivity towards SIRT2.^39,40,42^ ICL-SIRT078 was found to have significant neuroprotective effects in a lactacystin-induced model of Parkinsonian neuronal cell death in the N27 cell line. In addition, Rotili *et al.* synthesized tetracyclic pyrimidinedione derivatives obtained by cyclizing the 2-hydroxynaphthalene ring substituent of the core structure in Cambinol, and further opened a benzene ring to obtain a tricyclic pyrimidinedione derivatives. The resulted compounds 23 and 24 (Fig. 4) have improved inhibition against with SIRT2, but which exhibited potent SIRT1 inhibition.^43^

### 2.4 3-Benzenesulfonylaminobenzamide derivatives

**Fig. 5:**
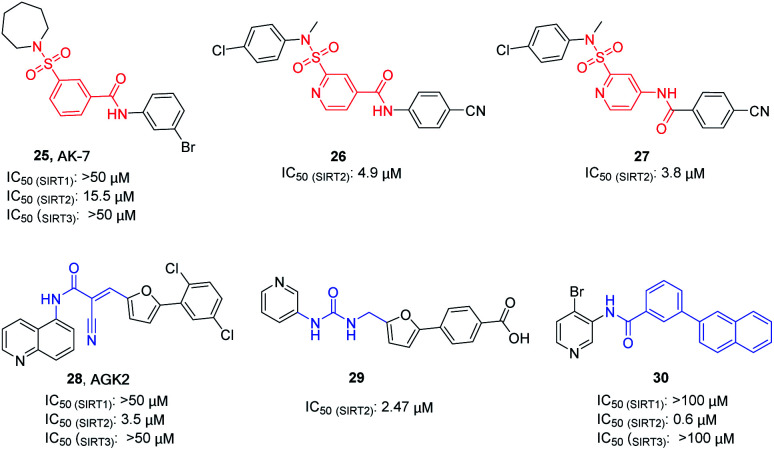
Chemical structures of 3-benzenesulfonylaminobenzamide-based SIRT2 inhibitors and their inhibitory activity data on SIRTs by Flour-de-Lys fluorescent assays.^45,46^

AK-7 (25, Fig. 5) is a representative 3-benzenesulfonamidophenyl SIRT2 inhibitor. Despite its weak inhibitory activity against SIRT2 (IC_50_ = 15.5 μM), researchers found that AK-7 has significant neuroprotective effects in the mouse model of Huntington's disease probably owing to its good brain permeability.^44^ To improve the potency of SIRT2 inhibitors containing the neuroprotective sulfobenzoic acid scaffold, Khanfar *et al.* designed, synthesized a compound library by changing the substituents of sulfonamide and benzoyl or inverting the amide group of benzamide, and screened the inhibition activity to SIRT2.^45^ The results showed that many compounds have improved activity compared with AK-7, of which 26 and 27 (Fig. 5) have the strongest inhibitory activity. AGK2 (28, Fig. 5) is another typical SIRT2 inhibitor.^20^ The structurally different AGK2 and AK-7 have been reported to exhibit good neuroprotective effects in neurodegenerative disease models. In 2007, Outeiro *et al.* reported that AGK2 can rescue α-synuclein toxicity and modified inclusion morphology in a cellular model of Parkinson's disease and protected against dopaminergic cell death both *in vitro* and in a Drosophila model of Parkinson's disease.^15,20^ Therefore, herein AGK2 is classified as the AK-7 class of inhibitors. Although AGK2 has obvious neuroprotective effect, the α,β-unsaturated carbonyl moiety of AGK2 may be related to possible off-target effects and unwanted toxicity. In order to improve the inhibitory activity and physicochemical properties of AGK2, our research group replaced the Michael receptor in AGK2 with different linkers and conducted preliminary structure–activity relationship study, leading to the identification of compound 29 (Fig. 5). Compared to AGK2, compound 29 exhibits 6–7 times higher potency (IC_50_ of 2.47 μM *vs.* 17.75 μM, measured under the same test condition).^46^ Moreover, Sakai *et al.* designed and synthesized a series of benzamide derivatives as SIRT2 inhibitors based on computational docking of AK-7 with SIRT2, as exemplified by compound 30 (Fig. 5), which has improved inhibitory activity and selectivity for SIRT2.^47^

### 2.5 Indole derivatives and their analogues

**Fig. 6:**
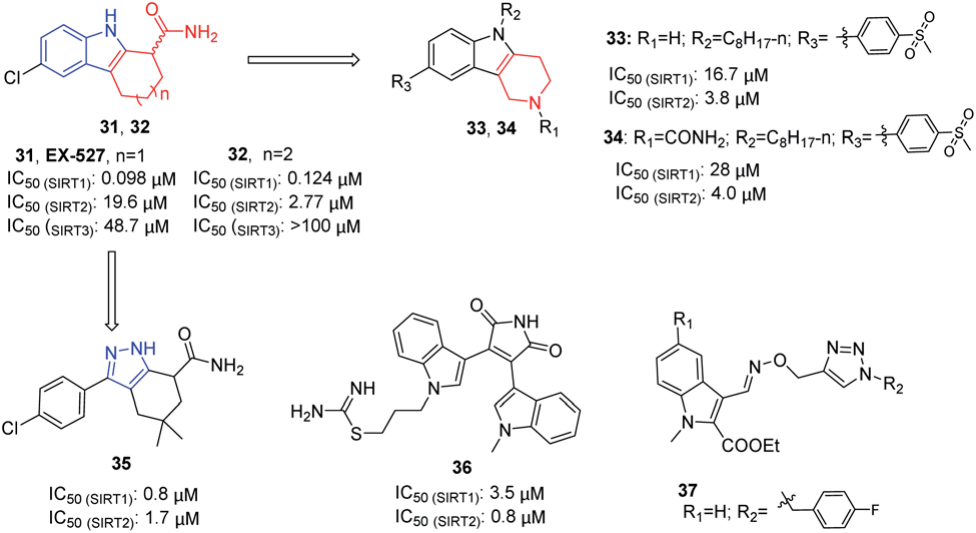
Chemical structures of indole-based SIRT2 inhibitors and their inhibitory activity data on SIRTs by isotope labelling,^49^ Sirt-Glo™ assay kit^50^ or fluorescence-based assays.^51^

In 2005, Napper *et al.* identified a series of indole compounds as SIRT inhibitors through high-throughput screening, *e.g.*31 and 32 (Fig. 6).^48^ These indole compounds have better inhibition against SIRT1 than SIRT2; kinetic analyses suggested that they exert inhibitory activity by preventing SIRT1-catalyzed deacetylation. To further explore the potential of the indole scaffold, Yang *et al.* designed and synthesized a set of new SIRT inhibitors by replacing the 2,3,4,9-tetrahydro-1*H*-carbazole core of EX-527 with 2,3,4,5-tetrahydro-1*H*-pyrido[4,3-*b*]indoles, for example 33 and 34 (Fig. 6).^49^ Compared to EX-527, 33 and 34 show improved inhibition and selectivity and significantly increase acetylation level of p53 and α-tubulin in HepG2 and MDA-MB-231 cells. In the same year, Therrien *et al.* designed and synthesized a series of 4,5,6,7-tetrahydro-1*H*-indazole derivatives by opening the benzene ring in 2,3,4,9-tetrahydro-1*H*-carbazole core and replacing the pyrrole with pyrazole. Among these derivatives, compound 35 (Fig. 6) has the best inhibitory activity on SIRT2, and moderate inhibitory effect on SIRT1.^50^ In 2006, Trapp *et al.* reported adenosine mimic-containing indoles as inhibitors of SIRTs, among which 36 (Fig. 6) inhibits SIRT1 and SIRT2 with IC_50_ values of 3.5 μM and 0.8 μM, respectively.^51^ Recently, Manjula *et al.* described a set of indole derivatives with an additional triazole moiety that can anchor the ligand in the binding cavity of SIRT1 and SIRT2, represented by 37 (Fig. 6).^52^

### 2.6 Tenovins and their analogues

**Fig. 7:**
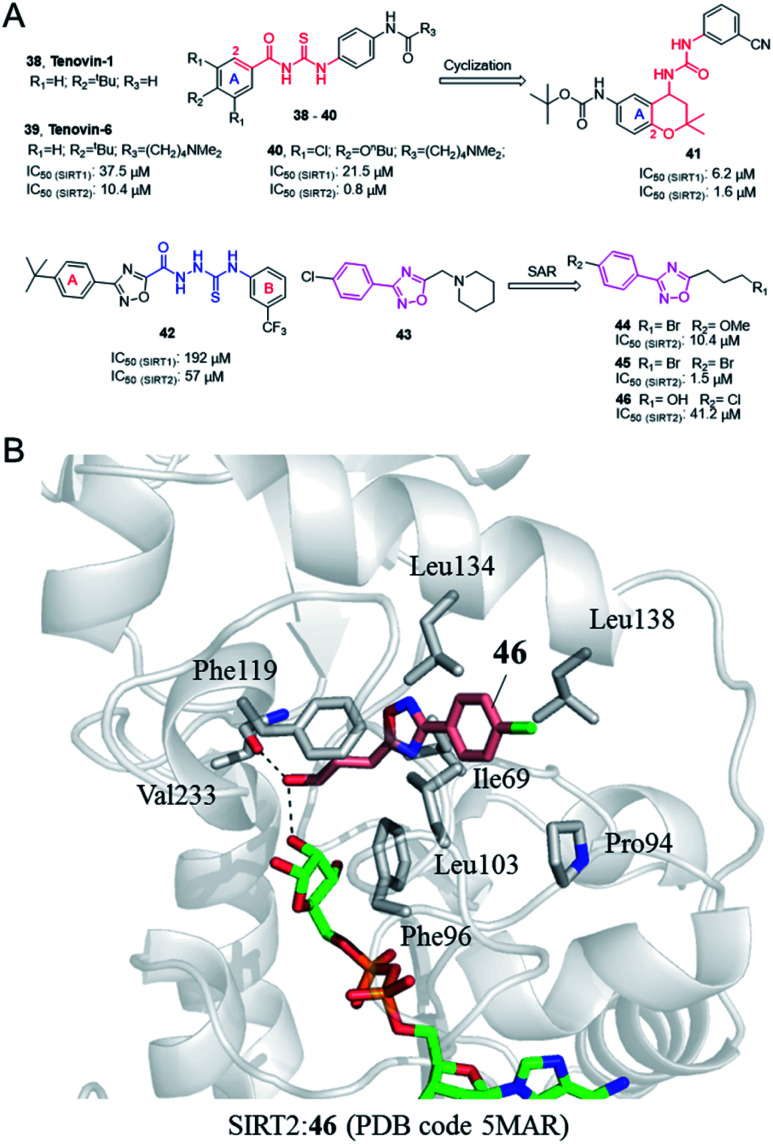
(A) Chemical structures of Tenovins and oxadiazoles-based SIRT2 inhibitors and their inhibitory activity data on SIRTs determined by coupled enzymatic deacetylation assay.^55^ (B) Crystallographic analyses revealed the binding features of 46 with SIRT2.^55^

In 2008, Lain *et al.* discovered the Sirtuin inhibitor Tenovin-1 (38, Fig. 7) by low-toxicity cell-based phenotypic screening; Tenovin-1 can activate the tumor suppressor p53 and inhibit tumor growth.^53^ Structural modifications led to a water-soluble analogue, Tenovin-6 (39, Fig. 7). Target validation studies revealed that Tenovins act through inhibition of deacetylation reaction catalyzed by SIRT1 and SIRT2. This group further conducted SAR studies on Tenovins, and synthesized a series of compounds by replacing A ring with different substituents. Among these compounds, compound 40 (Fig. 7) has significantly improved activity and selectivity compared with Tenovin-1 and Tenovin-6.

In recent years, Michael *et al.* established 1-(2,2-dimethyl-dihydrobenzopiperan-4-yl)-3-phenylurea derivatives as new SIRT1 and SIRT2 inhibitors, such as 41 (Fig. 6).^54^ Several compounds have obvious antiproliferative activity on U373, T98G and Hs683 glioma cells, and compound 41 also shows significant anticancer potential in zebrafish xenograft model. The 1-(2,2-dimethyl-dihydrobenzopiperan-4-yl)-3-phenylurea scaffold can be regarded as the cyclization of substitution in 2-position and the ortho carbonyl substituent of the A ring in the Tenovins to form a six-membered dimethyl pyran ring, accompanied by the replacement of thiourea with urea. Therefore, we assign this series of molecules as Tenovins analogues.

### 2.7 3,5-Disubstituted-1,2,4-oxadiazole derivatives

In 2008, Huhtiniemi *et al.* reported a lead compound 42 (Fig. 7A) containing 3,5-disubstituted-1,2,4-oxadiazole scaffold as a SIRT1 and SIRT2 inhibitor through virtual screening. Based on this hit compound, different substitutions and replacements of the benzene rings (A, B ring), and the modification of the linker between the oxadiazole ring and the B ring were made to obtain derivatives as SIRT inhibitor candidates. Among these derivatives, several compounds showed significantly improved SIRT1 inhibitory activity, but which did not show enhanced SIRT2 inhibition.^56^ Through virtual screening of a compound library into the peptide binding pocket of SIRT2, 3, 5 and 6, Steegborn *et al.* identified several potential ligands as SIRT2 inhibitors, such as 43 (Fig. 7A), which contains the 3,5-disubstituted-1,2,4-oxadiazole scaffold.^57^ Using 43 as the lead compound, a systematic SAR study was conducted, yielding a series of 3,5-disubstituted-1,2,4-oxadiazole derivatives as SIRT2 inhibitors with improved potency and selectivity, such as 44 and 45 (Fig. 7A).^58^ Kinetic studies indicated that the inhibition mechanism is likely uncompetitive towards both the peptide substrate and NAD^+^. The cellular results indicated that 44 and 45 induce apoptosis and show antiproliferative effects on the tested leukemia cells. Crystallographic studies of 46 in complex with SIRT2 revealed the binding in an unexplored selective hydrophobic site surrounded by Leu138, Leu134, and Ile69 (Fig. 7B), which further confirmed the specific hydrophobic site of SIRT2 for selective inhibitor development.

### 2.8 Benzopyrone derivatives

**Fig. 8:**
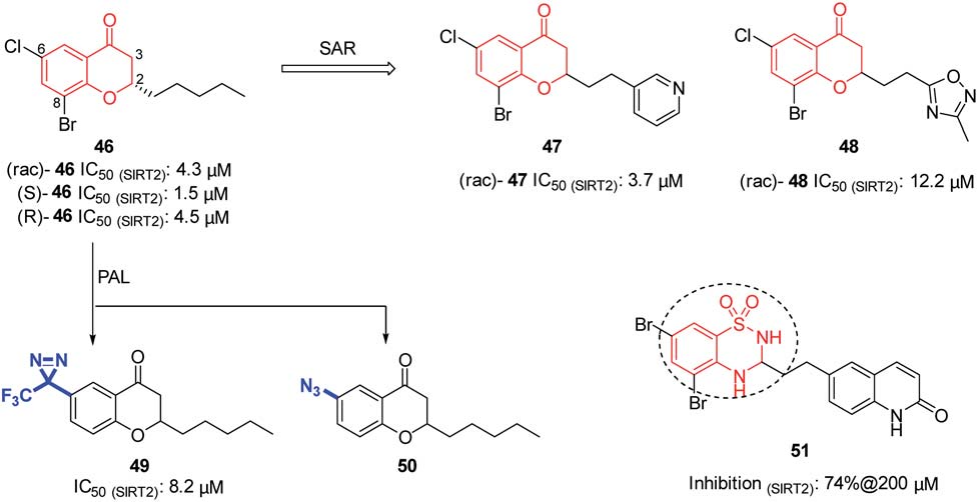
Chemical structures of benzopyrone-based SIRT2 inhibitors and their inhibitory activity data on SIRTs by Fluor-de-Lys or fluorescence-based assays.^59–62^

In 2012, Luthman' group reported the synthesis and evaluation of benzopyrone derivatives as selective SIRT2 inhibitors, such as 46 (Fig. 8).^59^ These derivatives were efficiently synthesized by base-mediated aldol condensation in a one-step process using microwave irradiation. Further SAR studies of benzopyrone scaffold focused on the effect of benzopyrone 2-position substituents on inhibitory activity.^60^ The activity of resulted compounds 47 and 48 (Fig. 8) is comparable or slightly lower than that of the racemic compound 46. Antiproliferative studies showed that 47 and 48 dose-dependently inhibit the proliferation of breast cancer MCF-7 cells and lung cancer A549 cells, and increase the acetylation level of α-tubulin in MCF-7 cells. Moreover, Luthman' group reported on the use of photoaffinity labeling (PAL) to identify the binding site of benzopyrone-based SIRT2 selective inhibitors in 2016.^61^ The photoactive active diazine and azide group were introduced at the 6-position of compound 46, resulting in photoactivatable probes 49 and 50 (Fig. 8). Compound 49 showed better inhibitory activity against SIRT2, which was used in subsequent PAL experiments to verify the binding site of benzopyrone derivatives in SIRT2. In this year, Luthman' group reported the exploration of bioisosteric replacement of benzopyrone core structure with different bicyclic scaffolds, including quinolin-4(1*H*)-ones, benzothiazine-1,1-dioxides, benzothiadiazine-1,1-dioxides and saccharins, resulting in various new derivatives.^62^ Biochemical assays showed that benzothiadiazine-1,1-dioxide derivatives have the strongest inhibitory activity against SIRT2, such as compound 51 (Fig. 8)

### 2.9 The others

**Fig. 9:**
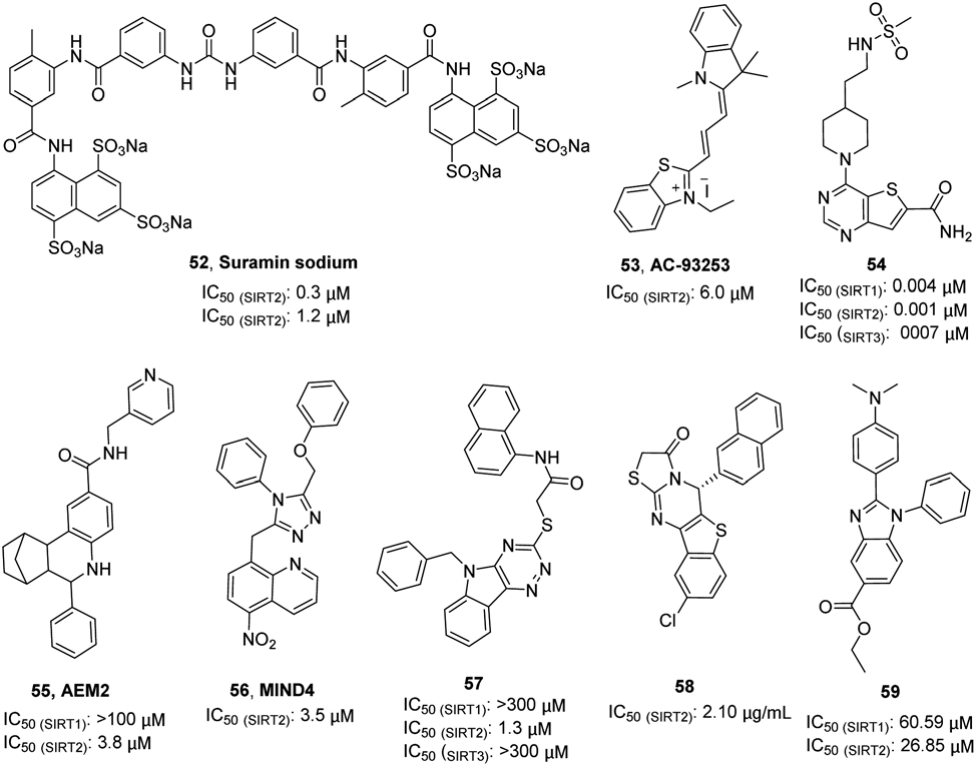
Chemical structures of other SIRT2 inhibitors and their inhibitory activity data on SIRTs.^63–68^

Suramin sodium (52, Fig. 9), a hydrophilic molecule with a molecular weight of over 1000, was reported by Trapp *et al.* in 2007 as a dual SIRT1/SIRT2 inhibitor.^63^ At the same year, Schuetz *et al.* identified suramin as a SIRT5 inhibitor and obtained the crystal structure of SIRT5 in complex with suramin, explaining inhibition mechanism suramin.^64^ In 2009, Zhang *et al.* reported a quaternary ammonium salt compound AC-93253 (53, Fig. 9) containing benzothiazole as a selective SIRT2 inhibitor.^65^ AC-93253 can significantly increase the histone acetylation level of α-tubulin, p53, and histone H4 in the cells, and exhibits selective cytotoxicity towards cancer cells by inducing apoptosis. In 2013, Disch *et al.* used Encoded Library Technology (ELT) to screen a 1.2 million heterocycle enriched library of DNA encoded small molecules and conducted SAR studies, identifying a set of pyrimidothiophene derivatives as pan-inhibitors of SIRT1/2/3 with nanomolar potency, such as 54 (Fig. 9). In 2014, Hoffmann *et al.* identified AEM2 (55, Fig. 9) as a moderate and selective SIRT2 inhibitor, which decreases SIRT2-dependent p53 deacetylation and thus sensitizes non-small cell lung cancer cell lines towards induction of apoptosis by the DNA damaging agent etoposide.^19^ The triazole derivative MIND4 (56, Fig. 9) was reported as a new SIRT2 inhibitor by Quinti *et al.* in 2016, and this inhibitor has neuroprotective activity in the Huntington's disease model of Drosophila.^66^ In 2017, Huang *et al.* found that compound 57 (Fig. 9) has good inhibitory activity on SIRT2 (IC_50(SIRT2)_ = 1.3 μM) and selectivity (IC_50(SIRT1)_ >300 μM, IC_50(SIRT3)_> 300 μM) through docking and SAR analysis.^67^ Recently, Khalil *et al.* designed and synthesized a series of benzothieno[3,2-*d*]pyrimidine derivatives, such as 58 (Fig. 9).^68^ Most of these compounds showed good cytotoxicity to breast cancer cell MCF-7 and renal cancer cell UO-31, and exhibited inhibition and selectivity for SIRT2. Besides, benzimidazole derivatives have been also reported as SIRT2 inhibitors, such as 59.^69–71^ These studies provided structurally diverse inhibitors to probe SIRT2 associated functions and diseases.

## 3. Perspectives

As an important member of the SIRT family, SIRT2 is highly homologous to SIRT1 and SIRT3, containing a smaller Zn^2+^ binding domain and a relatively larger Rossmann folding domain, and a catalytic core domain (including the substrate binding site and NAD^+^ binding site) formed by the “loop” connecting these two domains.^21^ While SIRT2 has a specific, deep hydrophobic site to accommodate long-chain acyl-lysine substrates as well as to recognize selective small-molecule inhibitors. As summarized above, several structurally distinct selective small-molecule SIRT2 inhibitors have been developed, which were demonstrated by crystallographic analyses to bind with the unique hydrophobic site of SIRT2, providing important clues or inspiration idea for future inhibitor design. Although no clinically useful SIRT2 inhibitors are available at present, the potential of selective SIRT2 inhibitors has been validated *in vitro* and *in vivo*, particularly with regard to their effectiveness in mediating or balancing related biological responses. The discovery and optimization strategies for SIRT2 inhibitors highlighted herein are expected to offer useful information and guidance to inhibitor development targeting SIRT2 and related target proteins.

## Conflicts of interest

There are no conflicts to declare.

## Supplementary Material
